# Examining the eye

**Published:** 2019-12-17

**Authors:** Allen Foster, Priya Morjaria

**Affiliations:** 1 Co-Director: International Centre for Eye Health, London School of Hygiene & Tropical Medicine, UK.; 2 Assistant Professor and Public Health Optometrist: London School of Hygiene & Tropical Medicine, UK.

**All health workers should be able to carry out a good eye examination of the front of the eye. In this issue, we explain how this can be done with limited resources.**

**Figure:**
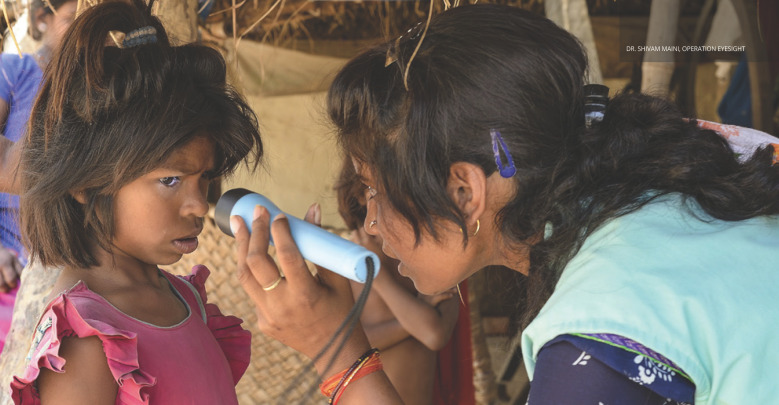
A number of common eye conditions can be detected using a torch. INDIA

Most patients with vision or eye problems will first be seen by a health worker who is not an ophthalmologist. In high-resource countries, this may be a general physician or an optometrist, and in low-resource settings it is more likely to be a community or primary health care worker. These health workers, who have to be able to assess any medical condition, often have limited knowledge and experience with regards to eye diseases, as well as limited equipment with which to examine the eye. This may result in health workers feeling disempowered and unable to help anyone with an eye condition. In practice, a number of common eye diseases can be diagnosed by examination of the eye with a torch and assessment of vision using a visual acuity chart, both of which are inexpensive and easy to use.

The aim of this issue is to support non-specialists to confidently carry out an eye examination.

The first step is to take a history. The presentation of common eye diseases can be usefully divided into four main groups of symptoms:

1. Red, sore, painful eye or eyes (including injury to the eye).
2. Decreased distance vision in one or both eyes, whether sudden or gradual.
3. A reduced ability to read small print or see near objects after the age of 40 years.
4. Any other specific eye symptom, such as double vision, swelling of an eyelid, watering or squint.

Deciding which of these main groups of symptoms a patient is complaining of enables us to start thinking about possible different diagnoses.

The second step is to measure the vision in each eye. This is described on page 46 for distance vision and on page 47 for those with difficulties to see for reading. Note: The severity of vision loss is an indicator of how serious the eye condition is.

The third step is to examine the front of the eye using a torch (p. 48). Ask:

- Are the eyes straight? Are the eyelids normal, and do they open and close? Are the eyelashes in place? Any swelling or redness?
- Is the white of the eye white? Any redness, discharge or swelling?
- Is the window of the eye (cornea), clear? Are there any grey or white areas?
- Is the pupil black and round, and does it become smaller in bright light? Is the red reflex present?

If the answer to these questions is ‘No’, then this can lead to a suspected diagnosis which may be treatable (such as conjunctivitis), or require referral (such as cataract).

There are other examinations that can also be performed with non-expensive equipment, including:

- Examining the optic nerve and retina using the Arclight (p. 49)
- Testing the red reflex (p. 53)
- Measuring intraocular pressure (p. 54)
- Examining visual fields (p. 56)
- Assessing eye alignment and movement (p. 58).

Not every health worker will have the knowledge, experience and equipment to perform all these further examinations; however we hope that this issue of the *Community Eye Health Journa*l will provide *all* health workers with the knowledge of how to take an eye history, measure visual acuity and perform a good examination of the external eye with a torch. We hope that this issue will provide you with the knowledge you need to feel confident in your work.

